# Magic wavelength for a rovibrational transition in molecular hydrogen

**DOI:** 10.1038/s41598-022-18159-y

**Published:** 2022-08-25

**Authors:** H. Jóźwiak, P. Wcisło

**Affiliations:** Institute of Physics, Faculty of Physics, Astronomy and Informatics, Nicolaus Copernicus University in Toruń, Grudziądzka 5, 87-100 Toruń, Poland

**Keywords:** Atomic and molecular physics, Electronic structure of atoms and molecules, Physics, Quantum mechanics

## Abstract

Molecular hydrogen, among other simple calculable atomic and molecular systems, possesses a huge advantage of having a set of ultralong living rovibrational states that make it well suited for studying fundamental physics. Further experimental progress will require trapping cold H_2_ samples. However, due to the large energy of the first electronic excitation, the conventional approach to finding a magic wavelength does not work for H_2_. We find a rovibrational transition for which the AC Stark shift is largely compensated by the interplay between the isotropic and anisotropic components of polarizability. The residual AC Stark shift can be completely eliminated by tuning the trapping laser to a specific “magic wavelength” at which the weak quadrupole polarizability cancels the residual dipole polarizability.

Accurate spectroscopy of simple calculable atomic and molecular systems has proven its importance for studying fundamental physics and testing quantum theory. A particularly important role has been played by atomic hydrogen. In addition to its large contribution to the development and tests of quantum electrodynamics, accurate spectroscopy of atomic hydrogen provides the energy scale for ab initio quantum calculations (the Rydberg constant) and gives an important contribution to the global adjustment of fundamental constants^1^. Several other calculable systems, such as helium atom^2,3^, HD^+^ ion^4,5^, exotic atoms^6–8^ or hydrogen molecule, contribute to testing quantum theory, determining fundamental constants and searching for new physics beyond the standard model^9–11^. When considering a long-term perspective, H_2_ possesses a huge advantage over other system, which is a set of a few hundred ultralong (a week) living rovibrational states^12^. The ratio of the natural linewidth to the optical transition frequency is on the order of $10^{-20}$ which, for a typical ability of resolving a $10^{-4}$ fraction of the linewidth, gives the ultimate limit on testing fundamental physics with H_2_ at $10^{-24}$ relative accuracy.

Fast progress in molecular hydrogen spectroscopy was triggered by implementing optical frequency combs over a decade ago. The present most accurate measurements were obtained with infrared-ultraviolet double resonance spectroscopy in molecular beam^13,14^ and cavity-enhanced spectroscopy: for HD, the sub-Doppler saturation technique was implemented^15,16^, while for homonuclear isotopologues, due to the lack of dipole transitions, Doppler-limited techniques were used^17,18^ (the Doppler-limited technique was also used for HD^19,20^). The highest accuracy, 13 kHz, was obtained for the HD isotopologue^13^. The factors that limit the accuracy depend on the approach used. For instance, for HD molecular beam experiments, the accuracy is limited by the residual first-order Doppler shift to the 12 kHz level^13^.

To maintain the fast progress in H_2_ rovibrational spectroscopy and progress towards the fundamental limitation, a cold H_2_ sample has to be trapped in an optical lattice. The first attempt to manipulate the H_2_ velocity with a laser field was demonstrated in Ref.^21^. Recent progress in laser technology already gives the capability to generate a 1 mK-deep optical-dipole trap with a continuous-wave (CW) laser coupled to a high-finesse cavity^22^. At this point, it is important to study this problem from the theory side and check if it is possible to eliminate the AC Stark effect caused by the trapping laser field. The conventional magic-wavelength approach^23^ is not applicable to the H_2_ molecule (see the next paragraph). Here, we demonstrate a new approach to finding a magic wavelength. First, we take advantage of the anisotropy of the dipole polarizability in H_2_ to eliminate the dominant part of the light shift by choosing an excited state with a favorable spatial orientation. Second, we calculate that the residual light shift can be completely eliminated by tuning the trap laser close to one of the rovibrational quadrupole transitions. We consider the S(0) 1–0 line in the H_2_ isotopologue. For this line, the magic wavelength is 2413 nm (0.23 MHz red detuned from the center of the Q(2) 1–0 line).

## Dipole polarizability (the interplay between the isotropic and anisotropic components)

**Figure 1:**
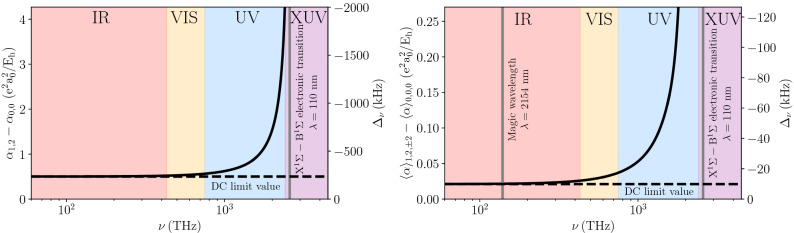
Difference between the dynamical polarizabilities in the excited, ${|v=1,N=2,M_{N}=\pm 2\rangle }$, and ground, ${|v=0,N=0,M_{N}=0\rangle }$, states (based on the data from Ref. H_2_^24^). The left panel shows the difference between the isotropic polarizabilities, while the right panel shows the difference between state-averaged polarizabilities. This plot involves only the dipole contribution to the polarizability. The secondary axis presents the light shift calculated for the trap depth of 0.12 mK, which corresponds to the trap laser intensity of 0.1 MW/mm^2^.

The isotropic dipole polarizability, α, of the H_2_ molecule in its ground electronic state, $X^{1}\Sigma _g^+$, is at the level of 5.4 e^2^a$_0^2/$E$_h$^24,25^, which for power densities achievable with today laser technology (1 MW/mm^2^ for a 0.4 mm laser beam waist)^22^ gives a depth of an optical dipole trap at the level of 1 mK. However, the polarizabilities in the vibrational ground, $v=0$, and first excited, $v=1$, states differ by almost 10%^25^; hence, the enormous light shift will ultimately dominate the uncertainty budget for the determination of the energies of the rovibrational transitions (the AC Stark effect not only shifts the effective position of a resonance but also causes its inhomogeneous broadening). The conventional approach to finding a magic wavelength used in atomic spectroscopy^23^ is not applicable to H_2_ molecule. The two spectroscopic states share the same electronic state; hence, the difference in polarizabilities changes very slowly with laser wavelength for the infrared and visible regions and is close to its DC value. The difference increases in the UV range^24,26^; see the left panel in Fig. 1. One may expect it to cross zero after the first electronic line, i.e., in the XUV range ($\lambda < 110$ nm), which is, however, difficult to access with available laser technology.

**Figure 2:**
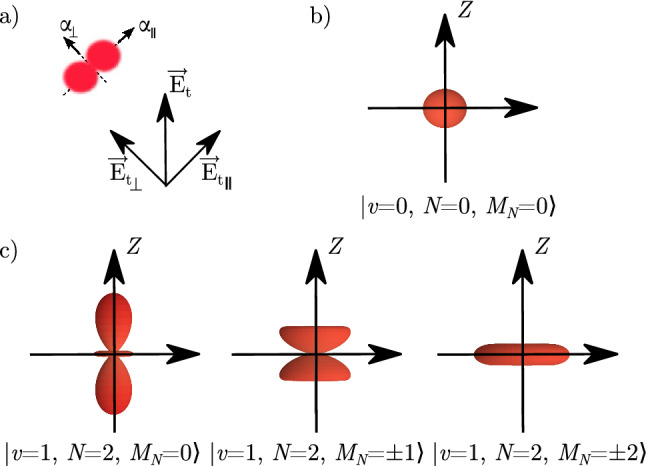
Orientation of the H$_{2}$ molecule with respect to the trapping field $\vec {E}_{t}$ and the definition of the parallel ($\alpha _{\parallel }$) and perpendicular ($\alpha _{\perp }$) components of the dipole polarizability (**a**). Panels (**b**, **c**) show the angle distribution of the H_2_ molecule in the ground and excited states, respectively (the squared modulus of $Y^{0}_{0}$ and $Y^{2}_{M_{N}}$ spherical harmonics), over which the two components of the polarizability are averaged to obtain $\langle \alpha \rangle _{v,N,M_N}$.

**Figure 3:**
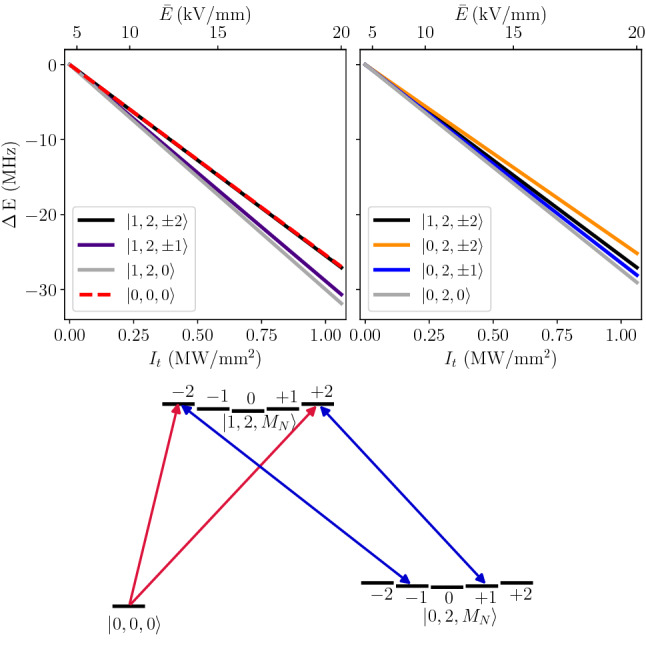
Stark shifts (the DC-limit values) of the $|v,N,M_{N}\rangle$ levels in H_2_: $|0,0,0\rangle$, and $|1,2,M_{N}\rangle$ (top left panel), and $|0,2,M_{N}\rangle$ and $|1,2,\pm 2\rangle$ (top right panel). The probe laser drives the $\Delta M_{N}=\pm 2$ components of the 1–0 S(0) transition (red arrows in the bottom panel), while the trapping laser couples the $|1, 2,\pm 2\rangle$ states with the $|0,2,\pm 1\rangle$ states, the two components of the 1–0 Q(2) line (blue arrows in the bottom panel).

For the case of the $X^{1}\Sigma _g^+$-state H_2_ molecule, we can benefit from the anisotropy of the dipole polarizability. When the electric field is aligned with the molecular axis, the static polarizability is larger by a quarter than α, and when the field is perpendicular, it is smaller by one eighth; the corresponding polarizability components are denoted by $\alpha _{\parallel }$ and $\alpha _{\perp }$; see Fig. 2a. The relative contribution of the two components to the effective polarizability in a given rovibrational state is determined by the direction of the trap laser electric field vector, $\vec {E}_t$, and the distribution of the molecular orientation in the laboratory frame, which is described by a specific spherical harmonic. We assume $\vec {E}_t$ to be directed along the *Z* axis in the laboratory frame. For cold *para*-H$_2$ experiments, one of the most preferable transitions to consider is the 1–0 S(0) line that links the $|v=0$, $N=0\rangle$ and $|v=1$, $N=2\rangle$ states; we focus the analysis on this transition. The distribution of the molecular orientation in these two states is given by $Y^{0}_{0}$ and $Y^{2}_{M_{N}}$ spherical harmonics; see Fig. 2b and c, respectively. For the ground state, the angle distribution is isotropic, and the corresponding average of $\alpha _{\parallel }$ and $\alpha _{\perp }$ simply gives $\alpha$. For the excited state, the angle distribution depends on the projection of the rotational angular momentum, $M_{N}$; see Fig. 2c. For $M_{N}=0$, a molecule is more likely to orient along the electric field, while for $M_{N}=\pm 2$, it is more likely to be orthogonal; hence, the corresponding average polarizability, $\langle \alpha \rangle$, is larger for $M_{N}=0$ and smaller for $M_{N}=\pm 2$. Direct integration of the two polarizability components over the angle distribution gives a general relation

1 $$\begin{aligned} \langle \alpha \rangle = \alpha - \frac{2}{3}\gamma \frac{3M_{N}^{2}-N(N+1)}{(2N-1)(2N+3)}, \end{aligned}$$

where $\alpha = \frac{1}{3}(\alpha _{\parallel } + 2\alpha _{\perp }$) and $\gamma = \alpha _{\parallel } - \alpha _{\perp }$ are called isotropic and anisotropic dipole polarizabilities. The Supplementary Information provides a general derivation of Eq. (1) based on the time-dependent perturbation theory and irreducible spherical tensor formulation; we further use this derivation for the determination of the dynamic quadrupole polarizability. The isotropic and anisotropic polarizabilities depend on the rovibrational state; hence, we label them $\alpha _{v,N}$ and $\gamma _{v,N}$. According to Eq. (1), the average value also depends on the $M_{N}$ number; hence, we label it $\langle \alpha \rangle _{v,N,M_{N}}$. The isotropic components in the ground and excited states differ by $9.3\%$, i.e., $\alpha _{0,0}=5.4179$ e$^2$a$_0^2/$E$_h$ and $\alpha _{1,2}=5.9193$ e$^2$a$_0^2/$E$_h$ (the numbers in this paragraph are the DC-limit values). It follows from Eq. (1), however, that the difference is reduced to below 0.4% when considering the $M_{N}=\pm 2$ angle distribution of the excited state, i.e., $\langle \alpha \rangle _{0,0,0}=\alpha _{0,0}=5.4179$ e$^2$a$_0^2/$E$_h$ and $\langle \alpha \rangle _{1,2,\pm 2}=\alpha _{1,2}-\frac{4}{21}\gamma _{1,2}=5.4390$ e$^2$a$_0^2/$E$_h$. In Fig. 1, the right panel, shows the polarizability difference for this transition as a function of laser frequency. The upper left panel in Fig. 3, shows the corresponding Stark shifts, $\Delta E^\mathrm{{dip}}_{v,N,M_{N}} = -(\bar{E}^{2}/2)\langle \alpha \rangle _{v,N,M_{N}}$, of the ground state and the three components of the excited state.

To drive the transitions from the ground $M_{N}=0$ to excited $M_{N}=\pm 2$ states, one should properly choose the polarization of the probe laser. The 1–0 S(0) transition is a quadrupole transition (the electric dipole transitions are not allowed in H_2_); hence, the light-molecule interaction does not probe the local electric field vector but the gradients of its components. Therefore, the selection rule does not involve only the relative orientation of the polarizations of the trap and probe lasers but also the direction of the probe laser propagation. The spherical components of the electric field gradient tensor corresponding to the $\Delta M_{N}=\pm 2$ transitions are (see the Supplementary Information for details)

2 $$\begin{aligned} \begin{aligned} \mathrm {T}&^{(2)}_{\pm 2} (\nabla \vec {{ E}}_{p}) =-\frac{(\partial _{X}E_{p,X}-\partial _{Y}E_{p,Y}) \pm i (\partial _{X}E_{p,Y} + \partial _{Y}E_{p,X})}{2\sqrt{6}}, \end{aligned} \end{aligned}$$

where $\vec {{ E}}_{p}$ is the probe laser electric field vector. Assuming that the wavelength is much shorter than the beam diameter, we can neglect the gradient components perpendicular to the direction of the probe laser propagation; hence, the $(\partial _{X}E_{p,X}-\partial _{Y}E_{p,Y})$ term can be neglected. For the assumed $\vec {E}_t$ directed along the *Z*-axis, a simple example of the configuration able to drive the $\Delta M_{N}=\pm 2$ transitions is $\vec {E}_p$ directed along the *X*-axis and propagating along the *Y*-axis. At this point, the selection rule does not depend on the direction of the trap laser propagation. It matters when considering the quadrupole contribution to the polarization; see the next paragraph. For that case, we assume the trap laser to propagate along the *Y*-axis (the same as for the probe laser).

**Figure 4:**
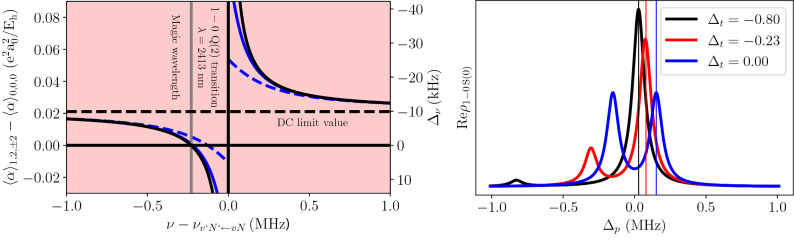
(Left panel) Difference between the average dynamical polarizabilities in the excited and ground states in H_2_. This figure shows the same quantity as the right panel Fig. 1, but also includes the quadrupole contribution and is zoomed around a close neighborhood of the 1–0 Q(2) line. In the simplest approach, the quadrupole contribution was calculated using perturbation theory (Eq. (3)), see the black solid line. Full calculations show that in a close neighborhood of a resonance, the quadrupole contribution to the polarizability depends on the intensity of a trap laser; for 1 MW/mm$^{2}$ the actual difference between the polarizabilities considerably deviates from the perturbation approach (see the blue dashed line), while for 0.1 MW/mm$^{2}$ the deviation is almost negligible (see the solid blue line). (Right panel) Shape of the 1–0 S(0) transition in H_2_ determined by solving a full master equation for a three-level system with two laser fields (see Supplementary Information) for different values of the detuning of the trapping laser, $\Delta _{t}$, and for $I_{t}=1$ MW/mm$^{2}$.

### Quadrupole polarizability: the magic wavelength

The difference in dynamic polarizabilities, shown in the right panel in Fig. 1, involves only the dipole contribution. Once the dominant part of the difference in dipole polarizability is eliminated by a proper choice of the ground and excited rovibrational states (see the red dashed and black solid lines in the upper left panel in Fig. 3), the quadrupole contribution is not negligible when the laser is tuned close to one of the rovibrational transitions. The dynamic quadrupole polarizability can be calculated as a sum over contributions from all rovibrational lines and electronic resonances. The electronic contribution to the quadrupole polarizability is negligible^27^. The quadrupole contribution to the polarizability is nonnegligible only when the trap laser is parked close to one of the rovibrational lines; in this regime, the contributions of all the other lines are completely negligible, and the full sum over the rovibrational transitions simplifies to

3 $$\begin{aligned} \begin{aligned} \langle \alpha ^\mathrm{{quad}}(\nu )\rangle _{vN} = \frac{k^{2}}{24h} (2N+1)(2N'+1) \begin{pmatrix} N &{} 2 &{} N' \\ 0 &{} 0 &{} 0 \end{pmatrix}^{2} \sum _{M_{N}'=-|N'|}^{N}\frac{|\langle v N|Q(r_{\mathrm {HH}}) |v'N'\rangle |^{2}}{\nu _{v'N'\leftarrow vN}-\nu } \Bigg |\sum _{p=\pm 1} \begin{pmatrix} N &{} 2 &{} N' \\ -M_{N} &{} p &{} M_{N}' \end{pmatrix}\Bigg |^{2}, \end{aligned} \end{aligned}$$

where $k=2\pi \nu /c$ is the magnitude of the wavevector of the trapping laser, *h* is the Planck constant, $\nu _{v'N'\leftarrow vN}$ denotes the frequency of the $v'N'\leftarrow vN$ rovibrational transition, $Q(r_\mathrm{{HH}})$ is the quadrupole transition moment function, which depends on the internuclear distance, $r_\mathrm{{HH}}$, and $$\begin{pmatrix} j_{1} &{} j_{2} &{} j \\ m_{1} &{} m_{2} &{} m \end{pmatrix}$$ is the Wigner 3-j symbol. In the Supplementary Information, we give a full derivation and a detailed discussion of Eq. (3). The left panel in Fig. 4 shows a close neighborhood of the Q(2) 1–0 line (at 2413 nm) that includes not only the dipole contribution to the polarizability difference, shown in the right panel in Fig. 1, but also the quadrupole contribution [given by Eq. (3)] due to the proximity of the Q(2) 1-0 line. Effectively, the difference in polarizabilities between the ground and excited states crosses zero at the point marked by the vertical gray line in the left panel in Fig. 4. At this wavelength, the total light shift vanishes, and following Refs.^23,28^, we refer to it as a *magic wavelength*. A major difference from a typical magic-wavelength approach is that in our case, the trap laser must be spectrally very narrow, and its absolute frequency must be well controlled, i.e., the value and dispersion of the trap laser frequency should be much smaller than 100 kHz. This is achievable with current optical frequency comb and ultrastable laser technologies.

**Table 1:** Magic wavelengths ($\lambda _{m}$) for the 1–0 S(0) transition calculated using time-dependent perturbation theory.

$\lambda _{m}$ (nm)	$\delta _{m}$ (MHz)	$\Omega$ (MHz)	Transition	$M_{N}'$	$\Delta _{\nu }$ (kHz)
1189	−0.10	0.014	2–0 S(0)	$\pm 1$	− 50
1207	0.08	0.016	3–1 S(2)	$\pm 3$/$\pm 1$	− 50/− 80
1318	0.04	0.012	3–1 Q(2)	$\pm 1$	70
2154	0.40	0.037	2–1 S(2)	$\pm 3$/$\pm 1$	− 32/− 57
2413	−0.23	0.028	1–0 Q(2)	$\pm 1$	8
2558	0.40	0.037	2–1 Q(2)	$\pm 1$	− 48
3003	−0.33	0.033	1–0 O(4)	$\pm 3$/$\pm 1$	− 2/14

In the second column ($\delta _{m}$) an accurate value of the magic wavelength is given respectively to the neighboring transition (specified in column 4). $\Omega$ is a Rabi frequency and $\Delta _{\nu }$ a light shift of the given $\Delta M_{N}$ component. $\Omega$ and $\Delta _{\nu }$ are calculated for the trap depth of 10 μK, which corresponds to the trap laser intensity of 8 kW/mm$^2$

The magic wavelength can be achieved by tuning the frequency of the trapping laser close to any of the rovibrational transitions involving either the ${|v=0,N=0,M_{N}=0\rangle }$ or ${|v=1,N=2,M_{N}=\pm 2\rangle }$ levels. Taking into account the selection rules associated with the 3-j symbols in Eq. (3) ($|N-2|\le N'\le N+2$, $N'=0\nleftrightarrow N=0$, and $M_{N}' = M_{N}\pm 1$) and the fact that there are 15 vibrational levels in H$_{2}$, we can identify 44 magic wavelengths for the 1–0 S(0) line. Table 1 lists the magic wavelengths that are the most beneficial from the perspective of experimental realization, i.e., the wavelengths are accessible with today laser technology and the magic wavelength detunings, $\delta _m$, and Rabi frequencies, $\Omega$, are the largest. Note that for transitions involving $N'=4$ levels there are two $M_{N}'$ components contributing to the sum in Eq. (3).

In a typical atomic dipole trap, the trap laser is far detuned from the nearest transition to avoid scattering losses^29^. Here this condition is followed, but the frequency regime is different. Instead of a strong and wide electronic line, we have an ultranarrow quadrupole rovibrational line; the few hundred kilohertz detuning is many orders of magnitude larger than the line width.

## The limitation of the perturbation approach

In this work, we consider very strong laser fields closely tuned to rovibrational resonance; hence, we should check if the perturbation approach [Eq. (3) and the black curve in the left panel in Fig. 4] is applicable. We do so by solving a full master equation for a three-level system interacting with two laser fields; see the Supplementary Information. In the extreme case (opposite to the perturbation approach regime), a strong trap laser is tuned to the resonance center, and the probe laser measures an Autler–Townes doublet; see the black curve in the right panel in Fig. 4. As the trap laser is getting detuned, one of the components becomes weaker, and effectively, for larger detunings, the doublet turns into a single line shifted from the unperturbed position, which reproduces the ordinary light shift described by perturbation theory [Eq. (3)]. The right panel in Fig. 4 shows the exact results of the polarizability difference calculated from the position of a dominant component of the Autler-Townes doublet (see Supplementary Information for details). The blue solid and dashed lines are for trap laser intensities of 0.1 and 1 MW/mm$^{2}$, respectively. These curves show that the resonant quadrupole contribution to the polarizability difference does not diverge to $\pm \infty$ at the line center as predicted by the perturbation approach, and hence, the corresponding light shift cannot be arbitrarily large. Furthermore, the intensity of the trap laser is limited by a demand that the polarizability should not depend on laser intensity (otherwise, only a fraction of the trapped molecules will be tuned to a magic wavelength). The left panel in Fig. 4 shows that this condition is fulfilled for trap laser intensities of 0.1 MW/mm$^{2}$ but not for 1 MW/mm$^{2}$. This limits the maximum depth of an optical dipole trap for which the magic wavelength can be applied; the trap depth corresponding to the intensity of 0.1 MW/mm$^{2}$ is 0.12 mK.

## Choice of a magic wavelength

At first glance, one could suspect that the most appropriate choice of a magic wavelength from the perspective of experimental realization would be either the 2–1 S(2) or 2–1 Q(2) line, since for these two cases the detuning of the trap laser is the largest and the strength of the trap laser-molecule coupling (expressed by the Rabi frequency $\Omega$) is the largest, as shown in Table 1. In this analysis, we should, however, take into account one more factor. The position of the trap laser resonance (an example of which is shown in Fig. 4) is also shifted by the strong trap laser field, i.e., the denominator in Eq. (3) also depends on the $M_N$ and $M'_N$ numbers. We should ensure that the light shift of the trap laser resonance is much smaller than the magic wavelength detuning, $\delta _{m}$. The last column in Table 1 shows the light shifts for each trap laser resonance for trap laser intensity corresponding to the trap depth of 10 $\mu$K. The table shows that the optimal choice of a magic wavelength is 2413 nm (− 0.23 MHz from the 1–0 Q(2) transition). For the trap depth of 10 *μ*K, the light shift of the 1–0 Q(2) line (the $M_N$ components marked with blue arrows in Fig. 3) is 8 kHz, which is over an order of magnitude smaller than the magic wavelength detuning $\delta _{m}=-0.23$ MHz.

## Outlook

We demonstrate a new approach to reducing the AC Stark shift for rovibrational lines in hydrogen molecule. We analyze the *para*-H$_2$ case; we identify a magic wavelength for the 1–0 S(0) at 2413 nm (−0.23 MHz from the 1–0 Q(2) transition). Important future directions include analysis of *ortho*-H$_2$ and other molecular hydrogen isotopologues that have nonzero nuclear spin and the corresponding hyperfine structure. The hyperfine structure will make the analysis more complex, but the much richer structure of states opens a perspective for identifying a combination of levels that is more beneficial from the perspective of experimental implementation of the magic wavelength.

## Supplementary Information

Supplementary Information.

## Data Availability

The datasets generated and analysed during the current study are available from the corresponding author on reasonable request.
