# Supplementary material for: Magic wavelength for a rovibrational transition in molecular hydrogen
Source: Sci Rep. 2022 Aug 25;12:14529. doi: 10.1038/s41598-022-18159-y (PMC9411631; doi:10.1038/s41598-022-18159-y)
Supplement: Supplementary file 1 — Supplementary Information. [file 41598_2022_18159_MOESM1_ESM.pdf]

Magic wavelength for a rovibrational transition in molecular hydrogen: supplementary information

H. Jóźwiak[1,*] and P. Wcisło[1]

[1]Institute of Physics, Faculty of Physics, Astronomy and Informatics, Nicolaus Copernicus University in Torun, Grudziadzka 5, 87-100 Toruń, Poland
[*]hubert.jozwiak@doktorant.umk.pl

ABSTRACT

This file provides the following supplementary information:
S1. Derivation of the dipole and quadrupole polarizability contribution to the Stark shift of rovibrational levels in H_2 using time-dependent perturbation theory
S2. Discussion of the selection rules for electric quadrupole transitions
S3. A non-perturbative analysis of the structure of rovibrational levels in H_2 in the presence of the trapping and probing laser beams, based on solution of the Liouville equation.

S1. H_2 molecule in external, time-dependent electric field: perturbation theory approach

In this section, we derive the formulas for the correction to the energy of rovibrational levels in the ground electronic $X^1\Sigma_g^+$ state in H_2 due to an external, time-dependent electric field. We use time-dependent perturbation theory up to the second order with respect to the amplitude of the perturbing electric field, following the formalism outlined by Sambe[1], and employ elements of the spherical tensor theory[2]. The derivation is based on the work of Gray and Lo[3], who introduced spherical tensor formalism to the theory of static molecular polarizabilities of Buckingham and coworkers[4] and a review on rotational and vibrational contribution to molecular polarizabilities and hyperpolarizabilities written by Bishop[5].

The Hamiltonian describing a molecule in an external, time-dependent electric field is a sum of the field-free molecular Hamiltonian, $\hat{H}_0$, and the perturbation, $\hat{H}'(t)$, which describes the interaction of molecular charges with external field

$$\hat{H}(t) = \hat{H}_0 + \hat{H}'(t). \tag{1}$$

The field-free molecular Hamiltonian yields the set of unperturbed eigenstates and energies

$$\hat{H}_0|\psi_n^{(0)}\rangle = E_n^{(0)}|\psi_n^{(0)}\rangle, \tag{2}$$

which will be specified later on. We consider the perturbation of molecular eigenstates due to the external, time-dependent, inhomogeneous electric field. The general form of the field is

$$\mathbf{E}(\mathbf{r},t) = \frac{\bar{E}}{\sqrt{2}}\left(\varepsilon e^{i(\mathbf{k}\cdot\mathbf{r}-2\pi\nu t)} + \varepsilon^* e^{-i(\mathbf{k}\cdot\mathbf{r}-2\pi\nu t)}\right), \tag{3}$$

where $\bar{E}$ is the mean electric field, related to the average intensity of the laser as $I_{\mathrm{av}} = c\varepsilon_0\bar{E}^2$, ε is a complex polarization vector of the propagating laser beam, ν is the frequency of the laser field and $\mathbf{k}$ denotes the wavevector with magnitude $k = |\mathbf{k}| = 2\pi\nu/c$. c and ε_0 denote the speed of light and vacuum permittivity, respectively.

The perturbation can be expressed as a Taylor series expansion with respect to a chosen position in the molecule[3,4]. Here, we restrict the expansion to the dipole and quadrupole terms

$$\hat{H}'(t) = -\sum_\sigma \hat{\mu}_\sigma E_\sigma(t) - \frac{1}{3}\sum_{\sigma,\tau}\hat{Q}_{\sigma\tau}\nabla E_{\sigma\tau}(t), \tag{4}$$

where the Greek subscripts σ, $\tau = X,Y,Z$ denote the Cartesian components. We use uppercase X,Y,Z symbols to refer to the components defined with respect to the space-fixed frame of reference, and lowercase x,y,z symbols to describe the components defined with respect to the molecule-fixed frame. E_σ and $\nabla E_{\sigma\tau}$ denote the components of the electric field and the electric field gradient at the origin of the expansion, respectively, $\hat{\mu}$ is the electric dipole moment operator

$$\hat{\mu}_\sigma = \sum_i q_i r_{i\sigma}, \tag{5}$$

where the sum goes over all molecular charges q_i and $r_{i\sigma}$ denotes the component of the position vector of the i-th charge. $\hat{Q}_{\sigma\tau}$ is the electric quadrupole moment operator, defined as

$$\hat{Q}_{\sigma\tau} = \frac{1}{2}\sum_i q_i(3r_{i\sigma}r_{i\tau} - r_i^2\delta_{\sigma\tau}). \tag{6}$$

Here, we assume that the electric field is linearly polarized in the space-fixed Z-direction, $\varepsilon = \varepsilon^* = \hat{\mathbf{Z}}$, and that the wavevector is oriented towards the space-fixed Y direction, $\mathbf{k} = k\hat{\mathbf{Y}}$. In such a case, the perturbation is given as

$$\hat{H}'(t) = -\frac{\bar{E}}{\sqrt{2}}\hat{\mu}_Z\Big(e^{-i2\pi\nu t} + e^{i2\pi\nu t}\Big) - \frac{ik\bar{E}}{3\sqrt{2}}\hat{Q}_{YZ}\Big(e^{-i2\pi\nu t} - e^{i2\pi\nu t}\Big), \tag{7}$$

It can be shown within the formalism of the time-dependent perturbation theory, that for the perturbation of the form

$$\hat{H}'(t) = \quad \hat{H}'_- e^{-i2\pi\nu t} + \hat{H}'_+ e^{i2\pi\nu t}, \tag{8}$$

there is no first-order correction to the eigenvalues of the unperturbed Hamiltonian, $\hat{H}_0$[1,6]. The second-order correction involves the cross products of $\hat{H}'_+$ and $\hat{H}'_-$

$$\Delta E_n^{(2)} = -\Big(\langle\psi_n^{(0)}|\hat{H}'_+\hat{\mathscr{R}}_n^+(\nu)\hat{H}'_-|\psi_n^{(0)}\rangle + \langle\psi_n^{(0)}|\hat{H}'_-\hat{\mathscr{R}}_n^-(\nu)\hat{H}'_+|\psi_n^{(0)}\rangle\Big), \tag{9}$$

where $\hat{\mathscr{R}}_n^{(\pm)}(\nu)$ is the frequency-dependent resolvent operator

$$\hat{\mathscr{R}}_n^{(\pm)}(\nu) = \sum_{n'}\frac{|\psi_{n'}^{(0)}\rangle\langle\psi_{n'}^{(0)}|}{E_{n'} - E_n \mp h\nu}. \tag{10}$$

In the case considered here the second-order correction is given as

$$\Delta E_n^{(2)} = -\frac{\bar{E}^2}{2}\Big(\langle\alpha_{ZZ}(\nu)\rangle_n - \langle C_{YZ,YZ}(\nu)\rangle_n\frac{k^2}{3}\Big), \tag{11}$$

where $\langle\alpha_{ZZ}(\nu)\rangle_n$ is the expectation value of the ZZ-component of the rank-2 dipole polarizability tensor, $\alpha_{\sigma\tau}(\nu)$, in the $|\psi_n^{(0)}\rangle$ state

$$\langle\alpha_{\sigma\tau}(\nu)\rangle_n = \sum_{n'}\Big(\frac{1}{E_{n'} - E_n - h\nu} + \frac{1}{E_{n'} - E_n + h\nu}\Big)\langle\psi_n^{(0)}|\hat{\mu}_\sigma|\psi_{n'}^{(0)}\rangle\langle\psi_{n'}^{(0)}|\hat{\mu}_\tau|\psi_n^{(0)}\rangle, \tag{12}$$

and $\langle C_{YZ,YZ}(\nu)\rangle_n$ denotes the expectation value of the YZ,YZ component of the rank-4 quadrupole polarizability tensor in the $|\psi_n^{(0)}\rangle$ state

$$\langle C_{\sigma\tau,\upsilon\phi}(\nu)\rangle_n = \frac{1}{3}\sum_{n'}\Big(\frac{1}{E_{n'} - E_n - h\nu} + \frac{1}{E_{n'} - E_n + h\nu}\Big)\langle\psi_n^{(0)}|\hat{Q}_{\sigma\tau}|\psi_{n'}^{(0)}\rangle\langle\psi_{n'}^{(0)}|\hat{Q}_{\upsilon\phi}|\psi_n^{(0)}\rangle. \tag{13}$$

Note that we do not consider the contribution from the rank-3 dipole-quadrupole polarizability tensor

$$\langle A_{\sigma,\tau\upsilon}(\nu)\rangle_n = \sum_{n'}\Big(\frac{1}{E_{n'} - E_n - h\nu} + \frac{1}{E_{n'} - E_n + h\nu}\Big)\langle\psi_n^{(0)}|\hat{\mu}_\sigma|\psi_{n'}^{(0)}\rangle\langle\psi_{n'}^{(0)}|\hat{Q}_{\tau\upsilon}|\psi_n^{(0)}\rangle, \tag{14}$$

since its expectation value vanishes for molecules with center of inversion, such as homonuclear diatomics[4,7].

The goal of this derivation is the correction to the energy of rovibrational states of *para*-H_2 in the ground electronic $X^1\Sigma_g^+$ state. The eigenstates $|\psi_n^{(0)}\rangle$ are written as a product of electronic, vibrational, and rotational eigenvectors

$$|\psi_n^{(0)}\rangle = |\eta\Lambda S\Sigma\nu;JM\Omega\rangle = |\eta\Lambda S\Sigma\rangle|\nu(J)\rangle|JM\Omega\rangle, \tag{15}$$

where $|\eta\Lambda S\Sigma\rangle$ denotes the electronic state of the diatomic molecule, $|\nu(J)\rangle$ is vibrational state (we explicitly display its dependence on the rotational quantum number due to centrifugal distortion), and $|JM\Omega\rangle$ is the angular (rotational) part of the molecular state. $\mathbf{S}$ and $\mathbf{L}$ denote the total electronic spin and the total electronic angular momentum, respectively, and Σ and Λ are the projections of $\mathbf{S}$ and $\mathbf{L}$ on the molecular axis. The angular momentum associated with the rotation of the nuclei is denoted as $\mathbf{R}$. $\mathbf{R}$ and $\mathbf{L}$ are coupled to form the intermediate angular momentum $\mathbf{N}$. Since $\mathbf{R}$ is perpendicular to the internuclear

axis, the projection of **N** on this axis is Λ. The total angular momentum (excluding nuclear spin, which is zero for *para*-H_2) is denoted as $\mathbf{J} = \mathbf{N} + \mathbf{S} = \mathbf{R} + \mathbf{L} + \mathbf{S}$, and its projection on the internuclear axis is $\Omega = \Lambda + \Sigma$. In the absence of external fields the square of the total angular momentum and the space-fixed Z-component of the total angular momentum, commute with the Hamiltonian. The projection of **J** on the space-fixed axis is denoted as M. The space-fixed projections of **R**, **L**, **N** and **S** are denoted as M_R, M_L, M_N, M_S, respectively. The rotational part, $|JM\Omega\rangle$, is the Wigner $\mathscr{D}$-function

$$\sqrt{\frac{2J+1}{8\pi^2}}\mathscr{D}^{J*}_{M\Omega}(\zeta), \tag{16}$$

where ζ describes the set of Euler angles describing the orientation of the molecule-fixed reference frame relative to the space-fixed frame. For the ground electronic $X^1\Sigma_g^+$ state of H_2, $S = 0$, $\Sigma = 0$, $\Lambda = 0$, and the notation is further simplified by putting $J = N$, $M = M_N$ and $\Omega = \Lambda = 0$. The rovibrational levels of *para*-H_2 in the $X^1\Sigma_g^+$ electronic states are thus denoted as

$$|\psi^{(0)}_{0,vNm_N}\rangle = |X^1\Sigma_g^+ v; NM_N\rangle = |X^1\Sigma_g^+\rangle|v(N)\rangle|NM_N\rangle, \tag{17}$$

where the rotational part, Eq. (16), is simplified to a spherical harmonic

$$\sqrt{\frac{2N+1}{8\pi^2}}\mathscr{D}^{N*}_{M_N,0} = \frac{1}{\sqrt{2\pi}}Y_{NM_N}, \tag{18}$$

denoted as $|NM_N\rangle$, and $|v(N)\rangle$ is the solution of the radial rovibrational Schrödinger equation, given, in the coordinate representation as $\chi_{vN}(r_{\mathrm{HH}})/r_{\mathrm{HH}}$, where r_{HH} is the internuclear distance in H_2. Substitution of Eq. (17) into Eqs. (12) and (13), leads to

$$\begin{aligned}\langle\alpha_{ZZ}(\nu)\rangle_{X^1\Sigma_g^+,vNM_N} = &\sum_{\eta'\Lambda'v'J'M'}\left(\frac{1}{E_{\eta'\Lambda'v'J'} - E_{X^1\Sigma_g^+vN} - h\nu} + \frac{1}{E_{\eta'\Lambda'v'J'} - E_{X^1\Sigma_g^+vN} + h\nu}\right)\\ &\times\left|\langle\eta'\Lambda'S\Sigma v'; J'M'\Omega'|\hat{\mu}_Z|X^1\Sigma_g^+ v; NM_N\rangle\right|^2,\end{aligned} \tag{19}$$

$$\begin{aligned}\langle C_{YZ,YZ}(\nu)\rangle_{X^1\Sigma_g^+,vNM_N} = &\frac{1}{3}\sum_{\eta'\Lambda'v'J'M'}\left(\frac{1}{E_{\eta'\Lambda'v'J'} - E_{X^1\Sigma_g^+vN} - h\nu} + \frac{1}{E_{\eta'\Lambda'v'J'} - E_{X^1\Sigma_g^+vN} + h\nu}\right)\\ &\times\left|\langle\eta'\Lambda'S\Sigma v'; J'M'\Omega'|\hat{Q}_{YZ}|X^1\Sigma_g^+ v; NM_N\rangle\right|^2,\end{aligned} \tag{20}$$

respectively. Note that as the electric dipole and quadrupole moment operators do not couple states with different S and Σ, these quantum numbers are omitted in the sum over primed states. As a consequence, the sum over $\Omega' = \Lambda' + \Sigma$ is also not written explicitly. In order to evaluate the matrix elements of the space-fixed components of the dipole and quadrupole moment operator in the basis introduced in Eq. (17), we express $\hat{\mu}_Z$ and $\hat{Q}_{YZ}$ as p-labelled space-fixed components of irreducible spherical tensor operators of rank 1 and 2, respectively[8]

$$\begin{aligned}\hat{\mu}_Z &= \mathrm{T}^{(1)}_{p=0}(\hat{\mu}),\\ \hat{Q}_{YZ} &= i\sqrt{\frac{3}{8}}\left(\mathrm{T}^{(2)}_{p=1}(\hat{\mathbf{Q}}) + \mathrm{T}^{(2)}_{p=-1}(\hat{\mathbf{Q}})\right),\end{aligned} \tag{21}$$

We express the space-fixed elements of the spherical tensors a combination of the q-labelled molecule-frame components and the Wigner $\mathscr{D}$-matrix

$$\begin{aligned}\mathrm{T}^{(1)}_{p=0}(\hat{\mu}) &= \sum_{q=-1}^{1}\mathscr{D}^{1*}_{0,q}(\zeta)\mathrm{T}^{(1)}_q(\hat{\mu}),\\ \mathrm{T}^{(2)}_{p=\pm1}(\hat{\mathbf{Q}}) &= \sum_{q=-2}^{2}\mathscr{D}^{2*}_{\pm1,q}(\zeta)\mathrm{T}^{(2)}_q(\hat{\mathbf{Q}}).\end{aligned} \tag{22}$$

We use a general formula for matrix elements of the $\mathscr{D}$-matrix,

$$\langle J',\Omega',M'|\mathscr{D}^{k*}_{pq}(\zeta)|J,\Omega,M\rangle = (-1)^{M'-\Omega'}\sqrt{(2J+1)(2J'+1)}\begin{pmatrix} J' & k & J\\ -M' & p & M\end{pmatrix}\begin{pmatrix} J' & k & J\\ -\Omega' & q & \Omega\end{pmatrix}, \tag{23}$$

to evaluate the matrix elements in Eqs. (19)-(20)

$$\langle J',\Omega',M'|\mathscr{D}^{1*}_{0q}(\zeta)|N,0,M_N\rangle = (-1)^{M'-\Omega'}\sqrt{(2N+1)(2J'+1)}\begin{pmatrix} J' & 1 & N \\ -M' & p & M_N \end{pmatrix}\begin{pmatrix} J' & 1 & N \\ -\Omega' & q & 0 \end{pmatrix}, \tag{24}$$

$$\langle J',\Omega',M'|\mathscr{D}^{2*}_{0q}(\zeta)|N,0,M_N\rangle = (-1)^{M'-\Omega'}\sqrt{(2N+1)(2J'+1)}\begin{pmatrix} J' & 2 & N \\ -M' & p & M_N \end{pmatrix}\begin{pmatrix} J' & 2 & N \\ -\Omega' & q & 0 \end{pmatrix}. \tag{25}$$

Here, $\begin{pmatrix} j_1 & j_2 & j \\ m_1 & m_2 & m \end{pmatrix}$, is the Wigner 3-j symbol which vanishes unless $m_1+m_2+m=0$. This implies that $M' = M_N + p$ and $q = \Omega'$. The sums in Eqs. (19)-(20) are then split into two terms, which involve couplings with the excited electronic states, and the couplings with rovibrational levels in the $X^1\Sigma_g^+$ state

$$\langle \alpha_{ZZ}(\nu)\rangle_{X^1\Sigma_g^+,vNM_N} = \langle \alpha^e_{ZZ}(\nu)\rangle_{X^1\Sigma_g^+,vNM_N} + \langle \alpha^{rv}_{ZZ}(\nu)\rangle_{X^1\Sigma_g^+,vNM_N}, \tag{26}$$

$$\langle C_{YZ,YZ}(\nu)\rangle_{X^1\Sigma_g^+,vNM_N} = \langle C^e_{YZ,YZ}(\nu)\rangle_{X^1\Sigma_g^+,vNM_N} + \langle C^{rv}_{YZ,YZ}(\nu)\rangle_{X^1\Sigma_g^+,vNM_N}. \tag{27}$$

S1.1 Dipole polarizability contribution to the Stark Shift

$\langle \alpha^e_{ZZ}(\nu)\rangle_{X^1\Sigma_g^+,vNM_N}$ denotes the contribution to the dipole polarizability from couplings with excited electronic states

$$\begin{aligned}\langle \alpha^e_{ZZ}(\nu)\rangle_{X^1\Sigma_g^+,vNM_N} =& (2N+1)\sum_{\eta'\Lambda'v'J'}\left(\frac{1}{E_{\eta'\Lambda'v'J'}-E_{X^1\Sigma_g^+vN}-h\nu}+\frac{1}{E_{\eta'\Lambda'v'J'}-E_{X^1\Sigma_g^+vN}+h\nu}\right)\\ &\times(2J'+1)\left|\langle\eta'\Lambda'v'(J')|\mathrm{T}^{(1)}_{q=\Omega'}(\mu)|X^1\Sigma_g^+v(N)\rangle\right|^2\begin{pmatrix} J' & 1 & N \\ -M_N & 0 & M_N \end{pmatrix}^2\begin{pmatrix} J' & 1 & N \\ -\Omega' & \Omega' & 0 \end{pmatrix}^2,\end{aligned} \tag{28}$$

where, due to symmetry properties of the 3-j symbols, Ω', and thus Λ', can take only three values: $-1,0,1$. Similar rules apply to J', which equals $N-1$, N and $N+1$. Hence, if the laser frequency is far detuned from the electronic resonance, the denominator can be approximated as

$$\frac{1}{E_{v'\eta'\Lambda'v'J'}-E_{X^1\Sigma_g^+vN}-h\nu} \approx \frac{1}{E_{v'\eta'\Lambda'v'N}-E_{X^1\Sigma_g^+vN}-h\nu}. \tag{29}$$

This leads to

$$\langle \alpha^e_{ZZ}(\nu)\rangle_{X^1\Sigma_g^+,vNM_N} = \langle \alpha^e_{\parallel}(\nu)\rangle_{X^1\Sigma_g^+,vN}\frac{2N^2+2N-2M_N^2-1}{(2N-1)(2N+3)} + \langle \alpha^e_{\perp}(\nu)\rangle_{X^1\Sigma_g^+,vN}\frac{N^2+N-M_N^2-1}{(2N-1)(2N+3)}, \tag{30}$$

where

$$\begin{aligned}\langle \alpha^e_{\parallel}(\nu)\rangle_{X^1\Sigma_g^+,vN} =& \sum_{\eta',\Lambda'=0,v'}\left(\frac{1}{E_{\eta'\Lambda'v'N}-E_{X^1\Sigma_g^+vN}-h\nu}+\frac{1}{E_{\eta'\Lambda'v'N}-E_{X^1\Sigma_g^+vN}+h\nu}\right)\\ &\times\left|\langle\eta'\Lambda'v'(J')|\mathrm{T}^{(1)}_{q=0}(\hat{\mu})|X^1\Sigma_g^+v(N)\rangle\right|^2,\end{aligned} \tag{31}$$

and

$$\begin{aligned}\langle \alpha^e_{\perp}(\nu)\rangle_{X^1\Sigma_g^+,vN} =& \sum_{\eta'\Lambda'=\pm1,v'}\left(\frac{1}{E_{\eta'\Lambda'v'N}-E_{X^1\Sigma_g^+vN}-h\nu}+\frac{1}{E_{\eta'\Lambda'v'N}-E_{X^1\Sigma_g^+vN}+h\nu}\right)\\ &\times\left|\langle\eta'\Lambda'v'(J')|\mathrm{T}^{(1)}_{q=\Lambda'}(\hat{\mu})|X^1\Sigma_g^+v(N)\rangle\right|^2,\end{aligned} \tag{32}$$

denote components of the dipole polarizability that are parallel and perpendicular to the molecular axis, respectively[9]. If we now introduce the isotropic and anisotropic parts of dipole polarizability as

$$\langle \alpha^e(\nu)\rangle_{X^1\Sigma_g^+,vN} = \frac{1}{3}(\langle \alpha^e_{\parallel}(\nu)\rangle_{X^1\Sigma_g^+,vN} + 2\langle \alpha^e_{\perp}(\nu)\rangle_{X^1\Sigma_g^+,vN}), \tag{33}$$

and

$$\langle \gamma^e(\nu)\rangle_{X^1\Sigma_g^+,vN} = \alpha^e_{\parallel}(\nu)\rangle_{X^1\Sigma_g^+,vN} - \langle \alpha^e_{\perp}(\nu)\rangle_{X^1\Sigma_g^+,vN}, \tag{34}$$

respectively, we obtain the electronic contribution to the dipole polarizability in a well-known form

$$\langle \alpha^{e}_{ZZ}(\nu)\rangle_{X^1\Sigma_g^+,vNM_N} = \langle \alpha^{e}(\nu)\rangle_{X^1\Sigma_g^+,vN} - \frac{2}{3}\langle \gamma^{e}(\nu)\rangle_{X^1\Sigma_g^+,vN}\frac{3M_N^2 - N(N+1)}{(2N-1)(2N+3)}. \tag{35}$$

One can similarly simplify the rovibrational contribution to the dipole polarizability, $\langle \alpha^{rv}_{ZZ}(\nu)\rangle_{X^1\Sigma_g^+,vNM_N}$, which involves couplings with rovibrational levels in the $X^1\Sigma_g^+$ state

$$\begin{aligned}\langle \alpha^{rv}_{ZZ}(\nu)\rangle_{X^1\Sigma_g^+,vNM_N} =&(2N+1)\sum_{v'}\sum_{N'}\Big(\frac{1}{E_{X^1\Sigma_g^+v'N'} - E_{X^1\Sigma_g^+vN} - h\nu} + \frac{1}{E_{X^1\Sigma_g^+v'N'} - E_{X^1\Sigma_g^+vN} + h\nu}\Big)\\ &\times (2N'+1)\left|\langle X^1\Sigma_g^+ v'(N')|\mathrm{T}^{(1)}_{q=0}(\hat{\mu})|X^1\Sigma_g^+ v(N)\rangle\right|^2 \begin{pmatrix} N' & 1 & N\\ -M_N & 0 & M_N\end{pmatrix}^2 \begin{pmatrix} N' & 1 & N\\ 0 & 0 & 0\end{pmatrix}^2.\end{aligned} \tag{36}$$

Since H_2 is a homonuclear molecule, the transition dipole moment in a given electronic state vanishes, and there is no contribution to the dipole polarizability due to $\langle \alpha^{rv}_{ZZ}(\nu)\rangle_{X^1\Sigma_g^+,vNM_N}$. The correction to the energy levels due to the dipole polarizability is thus given as

$$\Delta E^{\mathrm{dip}}_{X^1\Sigma_g^+,vNM_N} = -\frac{\bar{E}^2}{2}\left(\langle \alpha^{e}(\nu)\rangle_{X^1\Sigma_g^+,vN} - \frac{2}{3}\langle \gamma^{e}(\nu)\rangle_{X^1\Sigma_g^+,vN}\frac{3M_N^2 - N(N+1)}{(2N-1)(2N+3)}\right). \tag{37}$$

In the present work we use the rovibrationally-averaged values of the isotropic and anisotropic parts of the dynamical dipole polarizability tensor reported in Ref.[10]. Note that the authors provided the values of $\langle \alpha^{e}(\nu)\rangle_{X^1\Sigma_g^+,vN}$ and $\langle \gamma^{e}(\nu)\rangle_{X^1\Sigma_g^+,vN}$ for wavelengths in the range 182.25 – 1320.6 nm. For $\lambda > 1320.6$ nm we extrapolate the values from Ref.[10] using $y(\lambda) = a/(\lambda-\lambda_0)^b + y_{\mathrm{DC}}$, where y_{DC} is the DC-limit value of $\langle \alpha^{e}(\nu)\rangle_{X^1\Sigma_g^+,vN}$ or $\langle \gamma^{e}(\nu)\rangle_{X^1\Sigma_g^+,vN}$ reported in Ref.[10], and a, b, λ_0 are the fitting coefficients. In the article we use a simplified notation for the dipole correction to the energy of rovibrational levels in the ground electronic state of H_2

$$\Delta E^{\mathrm{dip}}_{vNM_N} = -\frac{\bar{E}^2}{2}\langle \alpha(\nu)\rangle_{vNM_N}, \tag{38}$$

with the term symbol of the electronic state made implicit for clarity.

S1.2 Quadrupole polarizability contribution to the Stark shift

In the case of the quadrupole polarizability, the contribution from the coupling with excited electronic states is given as

$$\begin{aligned}\langle C^{e}_{YZ,YZ}(\nu)\rangle_{X^1\Sigma_g^+,vNM_N} =&\frac{1}{8}(2N+1)\sum_{\eta'\Lambda'v'J'M'}\Big(\frac{1}{E_{\eta'\Lambda'v'J'} - E_{X^1\Sigma_g^+vN} - h\nu} + \frac{1}{E_{\eta'\Lambda'v'J'} - E_{X^1\Sigma_g^+vN} + h\nu}\Big)\\ &\times (2J'+1)\left|\langle \eta'\Lambda' v'(J')|\mathrm{T}^{(2)}_{q=\Omega'}(\hat{\mathbf{Q}})|X^1\Sigma_g^+ v(N)\rangle\right|^2 \begin{pmatrix} J' & 2 & N\\ -\Omega' & \Omega' & 0\end{pmatrix}^2\\ &\times\left(\delta_{M',M_N+1}\begin{pmatrix} J' & 2 & N\\ -M' & -1 & M_N\end{pmatrix}^2 + \delta_{M',M_N-1}\begin{pmatrix} J' & 2 & N\\ -M' & 1 & M_N\end{pmatrix}^2\right).\end{aligned} \tag{39}$$

The electronic contribution to the quadrupole polarizability in H_2 was studied in Ref.[11]. The authors provide the DC-limit values of the expectation values of the three independent components of the quadrupole polarizability tensor, defined in the molecule-fixed frame of reference, in several rovibrational levels of H_2. For instance, the DC-limit value of the $\langle C^{e}_{zz,zz}(\nu)\rangle_{X^1\Sigma_g^+,vNM_N}$ component, which involves the sum over $\left|\langle \eta'\Lambda' v'(J')|\mathrm{T}^{(2)}_{q=0}(\hat{\mathbf{Q}})|X^1\Sigma_g^+ v(N)\rangle\right|^2$ terms, is $6.518\,\mathrm{e^2a_0^4E_h^{-1}}$ and $7.729\,\mathrm{e^2a_0^4E_h^{-1}}$ for the $|v=0,N=0\rangle$ and $|v=1,N=2\rangle$ states in H_2, respectively[11]. For the wavelengths in the vicinity of $\lambda \approx 2.413\ \mu$m we are far detuned from electronic resonances, which occur at extreme UV (see Fig. 1 for the dependence of electronic dipole polarizability on the frequency of the electric field). We can thus assume that the electronic contribution to the dynamical quadrupole polarizability can be approximated by its DC counterpart. Substituting $k = 2\pi/\lambda$ to Eq. (11), we obtain the difference between the correction to the energy of the $|v=0,N=0\rangle$ and $|v=1,N=2\rangle$ states in H_2 due to the electronic part of the quadrupole polarizability to be at the level of $10^{-10}\,\mathrm{e^2a_0^2E_h^{-1}}$, and we neglect this contribution in the further analysis.

The correction to the energy levels due to the quadrupole polarizability is thus given only by the coupling with rovibrational levels of the $X^1\Sigma_g^+$ state

$$\Delta E^{\mathrm{quad}}_{X^1\Sigma_g^+,vNM_N} = -\langle C^{rv}_{YZ,YZ}(\nu)\rangle_{X^1\Sigma_g^+,vNM_N}\frac{k^2\bar{E}^2}{6}, \tag{40}$$

where

$$\begin{aligned}\langle C^{rv}_{YZ,YZ}(\nu)\rangle_{X^1\Sigma_g^+,vNM_N} =& \frac{1}{8}(2N+1)\sum_{v'N'}\left(\frac{1}{E_{X^1\Sigma_g^+v'N'}-E_{X^1\Sigma_g^+vN}-h\nu}+\frac{1}{E_{X^1\Sigma_g^+v'N'}-E_{X^1\Sigma_g^+vN}+h\nu}\right)\\ &\times(2N'+1)\left|\langle X^1\Sigma_g^+v'(N')|\mathrm{T}^{(2)}_{q=0}(\hat{\mathbf{Q}})|X^1\Sigma_g^+v(N)\rangle\right|^2\begin{pmatrix}N' & 2 & N\\ 0 & 0 & 0\end{pmatrix}^2\\ &\times\sum_{M'}\left(\delta_{M',M_N+1}\begin{pmatrix}N' & 2 & N\\ -M' & -1 & M_N\end{pmatrix}^2+\delta_{M',M_N-1}\begin{pmatrix}N' & 2 & N\\ -M' & 1 & M_N\end{pmatrix}^2\right).\end{aligned} \tag{41}$$

The sum over N' is restricted by the 3-j symbol to only three terms: $N'=N+2$ and $N'=N-2$, and $N'=N$, which correspond to the S, O and Q branches, respectively. The latter term vanishes if $v'=v$ (the pure rotational Q lines do not exist). Eq. (41) can be compared with Eq. (224) in Ref.[5], which presents the rovibrational contribution to the *static* quadrupole polarizability. Note that the authors consider the $C^{rv}_{ZZ,ZZ}$ component, for which the only non-vanishing element of the sum over M' involves the $M'=M_N$ term.

Here, we calculate the rovibrational contribution to the quadrupole field gradient polarizability using rovibrationally-averaged quadrupole transition moments reported in Ref.[12] and the transition frequencies for rovibrational transitions in H_2 obtained from the H2Spectre code of Czachorowski *et al*[13] and Komasa *et al.*[14]. In the article we use a simplified notation for the correction to the energy due to the quadrupole polarizability

$$\Delta E^{\mathrm{quad}}_{vNM_N} = -\frac{\bar{E}^2}{2}\langle\alpha^{\mathrm{quad}}(\nu)\rangle_{vNM_N}, \tag{42}$$

we drop the term symbol for the ground electronic state whenever possible, we introduce the frequency of each rovibrational resonance, $h\nu_{v'N'\leftarrow vN} = E_{X^1\Sigma_g^+v'N'}-E_{X^1\Sigma_g^+vN}$, and the quadrupole transition moment function $Q(r_{\mathrm{HH}})$[12]

$$\langle X^1\Sigma_g^+,v'N'|\mathrm{T}^{(2)}_{q=0}(\hat{\mathbf{Q}})|X^1\Sigma_g^+,vN\rangle = \langle X^1\Sigma_g^+,v'N'|\frac{1}{2}\sum_i q_i(3z_i^2-r_i^2)|X^1\Sigma_g^+,vN\rangle \equiv \langle v'N'|Q(r_{\mathrm{HH}})|vN\rangle. \tag{43}$$

If the frequency of the electric field is close to the resonant frequency $\nu_{v'N'\leftarrow vN}$, the rovibrational contribution in Eq. (41) can be approximated with a single term, and the correction to the quadrupole polarizability can be written as

$$\Delta E^{\mathrm{quad}}_{vNM_N} \approx \frac{\hbar|\Omega|^2}{4\Delta_t}, \tag{44}$$

where $\Delta_t = 2\pi(\nu-\nu_{v'N'\leftarrow vN})$ is the detuning from the resonance, and the Rabi frequency is defined as

$$\begin{aligned}|\Omega| =& \frac{\pi\bar{E}\nu}{\sqrt{3}c\hbar}\sqrt{(2N+1)(2N'+1)}\begin{pmatrix}N' & 2 & N\\ 0 & 0 & 0\end{pmatrix}\left|\langle v'N'|Q(r_{\mathrm{HH}})|vN\rangle\right|\\ &\times\sqrt{\sum_{M'}\left(\delta_{M',M_N+1}\begin{pmatrix}N' & 2 & N\\ -M' & -1 & M_N\end{pmatrix}^2+\delta_{M',M_N-1}\begin{pmatrix}N' & 2 & N\\ -M' & 1 & M_N\end{pmatrix}^2\right)}.\end{aligned} \tag{45}$$

S2. Selection rules for quadrupole transitions

The strength of the 1-0 S(0) electric quadrupole transition is related to the matrix element of the quadrupole term in the Hamiltonian that couples the initial and final spectroscopic states. Using irreducible spherical tensor operators, we can write

$$I \propto \langle v'=1, N'=2, M_N' | \mathrm{T}^{(2)}(\hat{\mathbf{Q}}) \cdot \mathrm{T}^{(2)}(\nabla \mathbf{E}) | v=0, N=0, M_N=0 \rangle, \tag{46}$$

an expand the scalar product in the space-fixed frame of reference

$$I \propto \sum_{p=-2}^{2} (-1)^p \langle v'=1, N'=2, M_N' | \mathrm{T}_p^{(2)}(\hat{\mathbf{Q}}) \mathrm{T}_{-p}^{(2)}(\nabla \mathbf{E}) | v=0, N=0, M_N=0 \rangle. \tag{47}$$

The space-fixed p-labelled components of the quadrupole moment are transformed to the molecule related to the q-labelled molecule-fixed components through the Wigner $\mathscr{D}$-matrix. Using Eq. (23) and the simplified notation introduced in Eq. (43), we obtain

$$\begin{aligned} I \propto \sum_{p=-2}^{2} (-1)^p \mathrm{T}_{-p}^{(2)}(\nabla \mathbf{E}) \sum_{q=-2}^{2} \langle v'=1, N'=2, M_N' | \mathscr{D}_{pq}^{(2)*} \mathrm{T}_q^{(2)}(\hat{\mathbf{Q}}) | v=0, N=0, M_N=0 \rangle = \\ = \langle v'=1, N'=2 | Q(r_{\mathrm{HH}}) | v=0, N=0 \rangle \left(\sum_{p=-2}^{2} (-1)^p \mathrm{T}_{-p}^{(2)}(\nabla \mathbf{E}) \begin{pmatrix} 2 & 2 & 0 \\ -M_N' & p & 0 \end{pmatrix} \right). \end{aligned} \tag{48}$$

The 3-j symbol indicates that the desired $\Delta M_N = \pm 2$ transition is driven by the $p = \pm 2$ components of the electric field gradient tensor. Moreover, the remaining two components of the 1-0 S(0) transition, $\Delta M_N = \pm 1$ and $\Delta M_N = 0$, can be eliminated if the electric field of the probe laser is oriented in such a way, that the $p = 0$ and $p = \pm 1$ components of the electric field gradient tensor vanish. The relation between spherical and Cartesian components of the field gradient, $\mathrm{T}_p^{(2)}(\nabla \mathbf{E})$ and $\partial_\alpha E_\beta$, is given as[3]

$$\begin{aligned} \mathrm{T}_0^{(2)}(\nabla \mathbf{E}) &= -\frac{1}{6}\Big(2\partial_Z E_Z - \partial_Y E_Y - \partial_X E_X\Big) \\ \mathrm{T}_{\pm 1}^{(2)}(\nabla E) &= \pm \frac{1}{2\sqrt{6}}\Big((\partial_X E_Z + \partial_Z E_X) \pm i(\partial_Y E_Z + \partial_Z E_Y)\Big) \\ \mathrm{T}_{\pm 2}^{(2)}(\nabla \mathbf{E}) &= -\frac{1}{2\sqrt{6}}\Big((\partial_X E_X - \partial_Y E_Y) \pm i(\partial_X E_Y + \partial_Y E_X)\Big). \end{aligned} \tag{49}$$

For the electric field of the form

$$\mathbf{E}(\mathbf{r}, t) = \frac{\xi}{\sqrt{2}}\Big(\varepsilon e^{i(\mathbf{k}\cdot\mathbf{r} - 2\pi v t)} + \varepsilon^* e^{-i(\mathbf{k}\cdot\mathbf{r} - 2\pi v t)}\Big), \tag{50}$$

the Cartesian elements of the electric field gradient tensor are simply

$$\partial_\alpha E_\beta = \frac{i\xi}{2}\mathbf{k}_\alpha\Big(\varepsilon_\beta e^{i(\mathbf{k}\cdot\mathbf{r} - 2\pi v t)} - \varepsilon_\beta^* e^{-i(\mathbf{k}\cdot\mathbf{r} - 2\pi v t)}\Big). \tag{51}$$

Thus, if the direction of propagation of the probe beam is set to the space-fixed Y axis, $\mathbf{k} = k\hat{\mathbf{Y}}$, and the probe beam is linearly polarized in the space-fixed X direction, the electric field vector is given as

$$\mathbf{E}(\mathbf{r}, t) = \frac{\xi}{2}\hat{\mathbf{X}}\Big(e^{i(kY - 2\pi v t)} + e^{-i(kY - 2\pi v t)}\Big), \tag{52}$$

the field gradient has only one non-zero Cartesian component

$$\partial_\alpha E_\beta = \delta_{\alpha Y}\delta_{\beta X}\frac{i\xi}{2}k\Big(e^{i(kY - 2\pi v t)} - e^{-i(kY - 2\pi v t)}\Big), \tag{53}$$

and two desired non-zero spherical components

$$\mathrm{T}_p^{(2)}(\nabla \mathbf{E}) = \mp \delta_{p \pm 2}\frac{\xi}{4}k\Big(e^{i(kY - 2\pi v t)} - e^{-i(kY - 2\pi v t)}\Big). \tag{54}$$

S3. H_2 molecule in external, time-dependent electric field: a non-perturbative approach based on the denisity matrix equation for a three-level system

The perturbation approach to the analysis of the rovibrational structure of the H_2 molecule in the external time-dependent electric field is limited to the frequency ranges that are significantly detuned from electronic or rovibrational resonances, $\Delta \gg \Omega$. The magic wavelength resulting from the cancellation of the residual dipole polarizability with the quadrupole polarizability is found for field frequencies detuned by less than 0.5 MHz from rovibrational resonances. Thus, we study the shape of the 1-0 S(0) resonance in the strong, external electric field in a non-perturbative way, based on the density matrix equation for the three-level system in a Λ-configuration, presented in Fig. 1. Level $|v=0, N=0, M_N=0\rangle \equiv |1\rangle$ is coupled with $|v=1, N=2, M_N=\pm 2\rangle \equiv |3\rangle$, level $|3\rangle$ is coupled with level $|v=0, N=2, M_N\rangle \equiv |2\rangle$, and there is no coupling between levels $|1\rangle$ and $|2\rangle$.

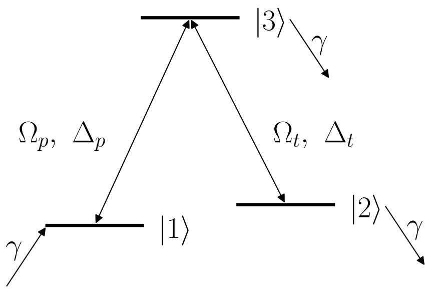

Figure 1. Configuration of the three-level system considered here.

The Hamiltonian for the system is a sum of the field-free part, $\hat{H}_0$, and the perturbation of the form introduced in Eq. (7), which involves the contributions from the two perturbing fields. The field that couples the $|1\rangle$ and $|3\rangle$ states and drives the 1-0 S(0) transition is linearly polarized in the X direction with $\mathbf{k}_1 = k_1\hat{\mathbf{Y}}$

$$\mathbf{E}_p(\mathbf{r},t) = \frac{\bar{E}_p}{\sqrt{2}}\hat{\mathbf{X}}\Big(e^{i(k_pY-\omega_pt)} + e^{-i(k_pY-\omega_pt)}\Big), \tag{55}$$

and the field that couples the $|2\rangle$ and $|3\rangle$ states (the trapping field) is linearly polarized in the Z direction with $\mathbf{k}_t = k_t\hat{\mathbf{Y}}$

$$\mathbf{E}_t(\mathbf{r},t) = \frac{\bar{E}_2}{\sqrt{2}}\hat{\mathbf{Z}}\Big(e^{i(k_tY-\omega_tt)} + e^{-i(k_tY-\omega_tt)}\Big). \tag{56}$$

Note that in this Section we use angular frequencies, $\omega = 2\pi\nu$, which are more convenient for solving the density matrix equations. The analysis in this Section is restricted to only three levels in the ground electronic state of H_2 – the dipole term in Eq. (7) is zero, and the three levels are coupled by non-diagonal elements of $\hat{H}'(t)$, which originate from the interaction of the molecular quadrupole moment with the electric field gradient. The Hamiltonian is thus given as

$$\hat{H}(t) = \hat{H}_0 + \hat{H}'(t) = \hbar\begin{pmatrix} 0 & 0 & \frac{1}{2}(\Omega_pe^{i\omega_pt} + \Omega_p^*e^{-i\omega_pt}) \\ 0 & \omega_{31}-\omega_{32} & \frac{1}{2}(\Omega_te^{i\omega_tt} + \Omega_t^*e^{-i\omega_tt}) \\ \frac{1}{2}(\Omega_pe^{i\omega_pt} + \Omega_p^*e^{-i\omega_pt}) & \frac{1}{2}(\Omega_te^{i\omega_tt} + \Omega_t^*e^{-i\omega_tt}) & \omega_{31}, \end{pmatrix} \tag{57}$$

where $\hbar\omega_{31} = E_3 - E_1$ and $\hbar\omega_{32} = E_3 - E_2$ are the (positive) frequencies of the 1-0 S(0) and 1-0 Q(2) resonances, and the Rabi frequencies are defined as

$$\Omega_p = \frac{2ik_p\bar{E}_p}{3\sqrt{2}\hbar}\langle 3|\hat{Q}_{YX}|1\rangle,\ \Omega_t = \frac{2ik_t\bar{E}_t}{3\sqrt{2}\hbar}\langle 3|\hat{Q}_{YZ}|2\rangle, \tag{58}$$

with the quadrupole moment operator, $\hat{\mathbf{Q}}$, defined in Eq. (6). The definition of the Rabi frequency for the trapping field is equivalent to Eq. (45). The frequencies of the lasers are assumed to be close to the corresponding resonances, hence, the we use the detunings from the unperturbed transition frequencies

$$\Delta_p = \omega_p - \omega_{31},\ \Delta_t = \omega_t - \omega_{32}. \tag{59}$$

The evolution of the three-level system is governed by the simplified Liouville equation

$$\dot{\hat{\rho}} = -\frac{i}{\hbar}\left[\hat{H},\hat{\rho}\right] - \hat{\Gamma}\hat{\rho}, \tag{60}$$

where $\hat{\Gamma}$ is a phenomenological operator describing the relaxation from the excited states and the damping of the coherences. Here, we assume that the rate of loss of the population in the excited states is given by γ, and the rate of loss of the coherences is $\Gamma = \frac{1}{2}\gamma$. Additionally, we assume that the same rate γ describes the gain of population in the ground $|1\rangle$ state. The relaxation term in Eq. (60) is thus given as

$$-\hat{\Gamma}\hat{\rho} = \begin{pmatrix} \gamma(\rho_{22}+\rho_{33}) & -\Gamma\rho_{12} & -\Gamma\rho_{13} \\ -\Gamma\rho_{21} & -\gamma\rho_{22} & -\Gamma\rho_{23} \\ -\Gamma\rho_{31} & -\Gamma\rho_{32} & -\gamma\rho_{33} \end{pmatrix}. \tag{61}$$

Eq. (60) involves six independent differential equations

$$\begin{aligned}
\dot{\rho}_{11} &= \gamma(\rho_{22}+\rho_{33}) - i(\rho_{31}-\rho_{13})(\bar{\Omega}_p e^{i\omega_p t} + cc.),\\
\dot{\rho}_{22} &= -\gamma\rho_{22} - i(\rho_{32}-\rho_{23})(\bar{\Omega}_t e^{i\omega_t t} + cc.),\\
\dot{\rho}_{33} &= -\gamma\rho_{33} + i(\rho_{31}-\rho_{13})(\bar{\Omega}_p e^{i\omega_p t} + cc.) + i(\rho_{32}-\rho_{23})(\bar{\Omega}_t e^{i\omega_t t} + cc.),\\
\dot{\rho}_{21} &= -(\Gamma + i(\omega_{31}-\omega_{32}))\rho_{21} + i\rho_{23}(\bar{\Omega}_p e^{i\omega_p t} + cc.) - i\rho_{31}(\bar{\Omega}_t e^{i\omega_t t} + cc.),\\
\dot{\rho}_{31} &= -(\Gamma + i\omega_{31})\rho_{31} + i(\rho_{33}-\rho_{11})(\bar{\Omega}_p e^{i\omega_p t} + cc.) - i\rho_{21}(\bar{\Omega}_t e^{i\omega_t t} + cc.),\\
\dot{\rho}_{32} &= -(\Gamma + i\omega_{32})\rho_{32} - i\rho_{12}(\bar{\Omega}_p e^{i\omega_p t} + cc.) + i(\rho_{33}-\rho_{22})(\bar{\Omega}_t e^{i\omega_t t} + cc.),
\end{aligned} \tag{62}$$

where, for brevity, half of the Rabi frequency is denoted as $\bar{\Omega}_n = \frac{1}{2}\Omega_n$. We are ultimately interested in the steady-state response of the three-level system, described by the off-diagonal elements, ρ_{21} and ρ_{32}. Following the standard analysis of the shape of the resonances through the density matrix equation[15,16], we represent the coherences as

$$\rho_{21} = \tilde{\rho}_{21} e^{-i(\omega_p-\omega_t)t},\ \rho_{32} = \tilde{\rho}_{32} e^{-i\omega_t t},\ \rho_{31} = \tilde{\rho}_{31} e^{-i\omega_p t}. \tag{63}$$

Inserting the new variables to the last three equations in (62) and invoking the rotating wave approximation (RWA), we obtain

$$\begin{aligned}
\dot{\tilde{\rho}}_{21} &= -(\Gamma - i(\Delta_p-\Delta_t))\tilde{\rho}_{21} + i\tilde{\rho}_{23}\bar{\Omega}_p^* - i\tilde{\rho}_{31}\bar{\Omega}_t,\\
\dot{\tilde{\rho}}_{31} &= -(\Gamma - i\Delta_p)\tilde{\rho}_{31} + i(\rho_{33}-\rho_{11})\bar{\Omega}_p^* - i\tilde{\rho}_{21}\bar{\Omega}_t^*,\\
\dot{\tilde{\rho}}_{32} &= -(\Gamma - i\Delta_t)\tilde{\rho}_{32} - i\tilde{\rho}_{12}\bar{\Omega}_p^* + i(\rho_{33}-\rho_{22})\bar{\Omega}_t^*.
\end{aligned} \tag{64}$$

In order to find the steady-state response of the three-level system, we invoke the slowly-varying envelope approximation (SVEA), we put $\dot{\tilde{\rho}}_{kl} = 0$, and we solve the coupled algebraic equations

$$\begin{aligned}
\tilde{\rho}_{21} &= L_{pt}\left(|\bar{\Omega}_p|\tilde{\rho}_{32}^* + |\bar{\Omega}_t|\tilde{\rho}_{31}\right),\\
\tilde{\rho}_{31} &= L_p\left(|\bar{\Omega}_p|(\rho_{33}-\rho_{11}) - |\bar{\Omega}_t|\tilde{\rho}_{21}\right),\\
\tilde{\rho}_{32} &= L_t\left(|\bar{\Omega}_t|(\rho_{33}-\rho_{22}) - |\bar{\Omega}_p|\tilde{\rho}_{21}^*\right),
\end{aligned} \tag{65}$$

where

$$L_p = (\Gamma - i\Delta_p)^{-1},\ L_t = (\Gamma - i\Delta_p)^{-1},\ L_{pt} = (\Gamma - i(\Delta_p-\Delta_t))^{-1}, \tag{66}$$

are complex Lorentzian functions associated with the detuning from the respective resonance, and where we used the fact that $\bar{\Omega}_n = i|\bar{\Omega}_n|$ (Eq. (58)). The steady-state SVEA coherences are eventually expressed as functions of steady-state populations

$$\begin{aligned}
\tilde{\rho}_{21} &= |\bar{\Omega}_p||\bar{\Omega}_t|\mathscr{C}\left(n_{32}L_t^* + n_{31}L_p\right),\\
\tilde{\rho}_{31} &= n_{31}|\bar{\Omega}_p|L_p\left(1 - |\bar{\Omega}_t|^2\mathscr{C}L_p\right) - n_{32}|\bar{\Omega}_p||\bar{\Omega}_t|^2 L_p L_t^*\mathscr{C},\\
\tilde{\rho}_{32} &= n_{32}|\bar{\Omega}_t|L_t\left(1 - |\bar{\Omega}_p|^2\mathscr{C}^* L_t\right) - n_{31}|\bar{\Omega}_p|^2|\bar{\Omega}_t|L_p^* L_t\mathscr{C}^*
\end{aligned} \tag{67}$$

where $n_{31} = \rho_{33} - \rho_{11}$, and $n_{32} = \rho_{33} - \rho_{22}$ are the population differences between the levels, and where

$$\mathscr{C} = \left(L_{pt}^{-1} + |\bar{\Omega}_p|^2 L_t^* + |\bar{\Omega}_t|^2 L_p \right)^{-1} \tag{68}$$

is introduced for the sake of brevity. The steady-state populations of the levels are found from the first three equations in (62), putting $\dot{\rho}_{kk} = 0$. Using additionally the condition

$$\rho_{11} + \rho_{22} + \rho_{33} = 1, \tag{69}$$

and the fact that the Rabi frequencies, as defined in Eq. (58), are purely imaginary, we obtain

$$\begin{aligned} \rho_{11} &= 1 + \frac{|\bar{\Omega}_p|}{\Gamma}\mathrm{Re}\tilde{\rho}_{31}, \\ \rho_{22} &= \frac{|\bar{\Omega}_t|}{\Gamma}\mathrm{Re}\tilde{\rho}_{32} \\ \rho_{33} &= -\frac{|\bar{\Omega}_p|}{\Gamma}\mathrm{Re}\tilde{\rho}_{31} - \frac{|\bar{\Omega}_t|}{\Gamma}\mathrm{Re}\tilde{\rho}_{32}. \end{aligned} \tag{70}$$

The real parts of the coherences from (67) are given as

$$\begin{aligned} \mathrm{Re}\tilde{\rho}_{31} &= \frac{n_{31}|\bar{\Omega}_p|\Gamma}{\Delta_p^2 + \Gamma^2}\left(1 - \alpha + \beta\frac{\Delta_p}{\Gamma}\right) - \frac{n_{32}|\bar{\Omega}_p|\Gamma}{\Delta_t^2 + \Gamma^2}\left(\alpha + \beta\frac{\Delta_t}{\Gamma}\right), \\ \mathrm{Re}\tilde{\rho}_{32} &= \frac{n_{32}|\bar{\Omega}_t|\Gamma}{\Delta_t^2 + \Gamma^2}\left(1 - \mathscr{A} - \mathscr{B}\frac{\Delta_t}{\Gamma}\right) - \frac{n_{31}|\bar{\Omega}_t|\Gamma}{\Delta_p^2 + \Gamma^2}\left(\mathscr{A} - \mathscr{B}\frac{\Delta_p}{\Gamma}\right), \end{aligned} \tag{71}$$

where we introduced

$$\begin{aligned} \alpha &= |\bar{\Omega}_t|^2 \mathrm{Re}(L_p\mathscr{C}),\ \beta = |\bar{\Omega}_t|^2 \mathrm{Im}(L_p\mathscr{C}), \\ \mathscr{A} &= |\bar{\Omega}_p|^2 \mathrm{Re}(L_t^*\mathscr{C}),\ \mathscr{B} = |\bar{\Omega}_p|^2 \mathrm{Im}(L_t^*\mathscr{C}). \end{aligned} \tag{72}$$

Substituting (71) to (70) leads to the final formula for the difference between the populations of the state $|1\rangle$ and $|3\rangle$

$$n_{31} = -\frac{1}{1 + \frac{1}{\Delta_p^2+\Gamma^2}\left(2|\bar{\Omega}_p|^2\left(1 - \alpha + \beta\frac{\Delta_p}{\Gamma} - (\alpha + \beta\frac{\Delta_t}{\Gamma})\mathscr{D}\right) - |\bar{\Omega}_t|^2\left(\mathscr{A} - \mathscr{B}\frac{\Delta_p}{\Gamma} - (1 - \mathscr{A} - \mathscr{B}\frac{\Delta_t}{\Gamma})\mathscr{D}\right)\right)}, \tag{73}$$

and for the difference between the populations of the state $|2\rangle$ and $|3\rangle$

$$n_{32} = n_{31}\left(\frac{\Delta_t^2 + \Gamma^2}{\Delta_p^2 + \Gamma^2}\right)\mathscr{D}, \tag{74}$$

where we have introduced

$$\mathscr{D} = \frac{2|\bar{\Omega}_t|^2(\mathscr{A} - \mathscr{B}\frac{\Delta_p}{\Gamma}) - |\bar{\Omega}_p|^2(1 - \alpha + \beta\frac{\Delta_p}{\Gamma})}{\Delta_t^2 + \Gamma^2 + 2|\bar{\Omega}_t|^2(1 - \mathscr{A} - \mathscr{B}\frac{\Delta_t}{\Gamma}) - |\bar{\Omega}_p|^2(\alpha + \beta\frac{\Delta_t}{\Gamma})}. \tag{75}$$

Note that such form of the formula for n_{31} is convenient if one wants to recover the population difference for the two-level system[16], by putting $\bar{\Omega}_t = 0$

$$n_{31} = -\left(1 + \frac{2|\bar{\Omega}_p|^2}{(\Delta_p^2 + \Gamma^2)}\right)^{-1}. \tag{76}$$

The explicit formulas for the coherences are found by substituting Eqs. (73) and (74) to (67). Of particular importance is the real part of the steady-state SVEA coherence between states $|1\rangle$ and $|2\rangle$, which determines the shape of the 1-0 S(0) transition induced by the probe laser. Substituting Eq. (74) to (71) leads to

$$\mathrm{Re}\tilde{\rho}_{31} = \frac{n_{31}|\bar{\Omega}_p|\Gamma}{\Delta_p^2 + \Gamma^2}\left(1 - \alpha(1 + \mathscr{D}) + \frac{\beta}{\Gamma}(\Delta_p - \Delta_t\mathscr{D})\right). \tag{77}$$

Similarly to Eq. (73), this particular form is convenient if one wants to recover the result for the two-level system

$$\mathrm{Re}\tilde{\rho}_{31} = \frac{n_{31}|\bar{\Omega}_p|\Gamma}{\Delta_p^2+\Gamma^2} = -\frac{|\bar{\Omega}_p|\Gamma}{\Delta_p^2+\Gamma^2+2|\bar{\Omega}_p|^2}, \tag{78}$$

which describes the power-broadened Lorentzian shape of an optical resonance.

Here, we determine the shape of the 1-0 S(0) resonance from Eq. (77) for various detunings of the trapping beam, Δ_t, assuming that the Rabi frequency of the probe beam is significantly smaller than the Rabi frequency of the trapping beam. The value of Γ is fixed at 0.1 MHz.

References

1. Sambe, H. Steady states and quasienergies of a quantum-mechanical system in an oscillating field. *Phys Rev A* **7**, 2203–2213, DOI: 10.1103/PhysRevA.7.2203 (1973).
2. Fano, U. & Racah, G. *Irreducible tensorial sets*. Cambridge Molecular Science (Academic Press Inc., 1959).
3. Gray, C. & Lo, B. Spherical tensor theory of molecular multipole moments and polarizabilities. *Chem. Phys.* **14**, 73–87, DOI: 10.1016/0301-0104(76)80028-6 (1976).
4. Buckingham, A. D. Permanent and induced molecular moments and long-range intermolecular forces. In *Adv Chem Phys*, 107–142, DOI: 10.1002/9780470143582.ch2 (John Wiley & Sons, Inc., 2007).
5. Bishop, D. M. Molecular vibrational and rotational motion in static and dynamic electric fields. *Rev Mod Phys* **62**, 343–374, DOI: 10.1103/RevModPhys.62.343 (1990).
6. Beloy, K. *Theory of the AC Stark effect on the atomic hyperfine structure and applications to microwave atomic clocks.* (University of Nevada, Reno, 2009).
7. McLean, A. D. & Yoshimine, M. Theory of molecular polarizabilities. *J Chem Phys* **47**, 1927–1935, DOI: 10.1063/1.1712220 (1967).
8. Brown, J. M. & Carrington, A. *Rotational Spectroscopy of Diatomic Molecules*. Cambridge Molecular Science (Cambridge University Press, 2003).
9. Krems, R. V. *Molecules in electromagnetic fields: from ultracold physics to controlled chemistry.* (John Wiley & Sons, Inc., 2018).
10. Raj, A., Hamaguchi, H. & Witek, H. A. Polarizability tensor invariants of H_2, HD, and D_2. *J Chem Phys* **148**, 104308, DOI: 10.1063/1.5011433 (2018).
11. Miliordos, E. & Hunt, K. L. C. Dependence of the multipole moments, static polarizabilities, and static hyperpolarizabilities of the hydrogen molecule on the H–H separation in the ground singlet state. *J Chem Phys* **149**, 234103, DOI: 10.1063/1.5066308 (2018).
12. Jóźwiak, H., Cybulski, H. & Wcisło, P. Hyperfine components of all rovibrational quadrupole transitions in the H_2 and D_2 molecules. *J Quant Spectrosc Radiat Transfer* **253**, 107186, DOI: https://doi.org/10.1016/j.jqsrt.2020.107186 (2020).
13. H2SPECTRE ver. 7.0. Fortran source code, 2019; P. Czachorowski, Ph.D. thesis, University of Warsaw, Poland, 2019.
14. Komasa, J., Puchalski, M., Czachorowski, P., Łach, G. & Pachucki, K. Rovibrational energy levels of the hydrogen molecule through nonadiabatic perturbation theory. *Phys Rev A* **100**, 032519, DOI: 10.1103/PhysRevA.100.032519 (2019).
15. May, A. D. Molecular dynamics and a simplified master equation for spectral line shapes. *Phys Rev A* **59**, 3495–3505, DOI: 10.1103/PhysRevA.59.3495 (1999).
16. Rand, S. C. *Lectures on light: nonlinear and quantum optics using the density matrix* (Oxford University Press, 216).
